# Morphological and Genetic Evidence for Multiple Evolutionary Distinct Lineages in the Endangered and Commercially Exploited Red Lined Torpedo Barbs Endemic to the Western Ghats of India

**DOI:** 10.1371/journal.pone.0069741

**Published:** 2013-07-22

**Authors:** Lijo John, Siby Philip, Neelesh Dahanukar, Palakkaparambil Hamsa Anvar Ali, Josin Tharian, Rajeev Raghavan, Agostinho Antunes

**Affiliations:** 1 Marine Biotechnology Division, Central Marine Fisheries Research Institute (CMFRI), Kochi, India; 2 Export Inspection Agency (EIA), Kochi, India; 3 CIMAR/CIIMAR, Centro Interdisciplinar de Investigação Marinha e Ambiental, Rua dos Bragas, Porto, Portugal; 4 Departamento de Biologia, Faculdade de Ciências, Universidade do Porto, Rua do Campo Alegre, Porto, Portugal; 5 Conservation Research Group (CRG), St. Albert’s College, Kochi, India; 6 Indian Institute of Science Education and Research (IISER), Pune, India; 7 Zoo Outreach Organization (ZOO), Coimbatore, India; 8 Department of Zoology, St. John’s College, Anchal, Kerala, India; 9 Durrell Institute of Conservation and Ecology (DICE), University of Kent, Canterbury, United Kingdom; 10 Research Group Zoology: Biodiversity & Toxicology, Center for Environmental Sciences, University of Hasselt, Diepenbeek, Belgium; Aberystwyth University, United Kingdom

## Abstract

Red lined torpedo barbs (RLTBs) (Cyprinidae: *Puntius*) endemic to the Western Ghats Hotspot of India, are popular and highly priced freshwater aquarium fishes. Two decades of indiscriminate exploitation for the pet trade, restricted range, fragmented populations and continuing decline in quality of habitats has resulted in their ‘Endangered’ listing. Here, we tested whether the isolated RLTB populations demonstrated considerable variation qualifying to be considered as distinct conservation targets. Multivariate morphometric analysis using 24 size-adjusted characters delineated all allopatric populations. Similarly, the species-tree highlighted a phylogeny with 12 distinct RLTB lineages corresponding to each of the different riverine populations. However, coalescence-based methods using mitochondrial DNA markers identified only eight evolutionarily distinct lineages. Divergence time analysis points to recent separation of the populations, owing to the geographical isolation, more than 5 million years ago, after the lineages were split into two ancestral stocks in the Paleocene, on north and south of a major geographical gap in the Western Ghats. Our results revealing the existence of eight evolutionarily distinct RLTB lineages calls for the re-determination of conservation targets for these cryptic and endangered taxa.

## Introduction

Of the 5±3 million species on earth, only 1.5 million have names [1]. Accelerating the description of unknown biodiversity continues to be a major challenge as extinction rates increase [2] and modern taxonomy is far from reaching a scientific consensus on species concept and delimitation [3], [4]. As a result, distinctive units, such as evolutionarily significant units (ESUs) or designatable units (DUs), which are appropriate targets for conservation, may remain undetected for long periods of time [5]. This is a critical impediment particularly for regions harboring exceptionally high biodiversity, that face a high risk of anthropogenic impacts [6] and also among speciose yet poorly known taxa, such as reptiles [7]–[9] and freshwater fishes [10]–[13].

The order Cypriniformes is a monophyletic group of primary freshwater fishes containing over 3500 species, with a wide distribution in North America, Europe, Africa and Asia [14], [15]. These fishes are an essential protein source for many societies, are highly valued in recreational fisheries and constitute a major component of the tropical fish trade [12]. Being a taxonomically diverse group exhibiting a remarkable and fascinating array of morphologies, cypriniform fishes present many challenges to systematists and evolutionary biologists [15], [16]. Such challenges are particularly severe in biogeographic ‘Hotspots’ such as the Western Ghats (WG) of India, where endemic lineages have evolved in several taxa due to extended geographical isolation [17]–[19].

Several small (<220 km) and isolated (not inter-connected) west flowing rivers between 8° and 12° latitudes in the WG harbor a unique assemblage of endemic freshwater fishes, sometimes as high as 129 species within a sub-basin [20]. This remarkable diversity is nevertheless known to be a gross under-representation [21], as around 10–20% of fish species in any basin of reasonable size in this region are likely to be undescribed [22]. Connections and divisions between rivers affect opportunities for dispersal, which while allowing the gene flow between some populations may promote the isolation of others [23], [24].

**Figure 1:**
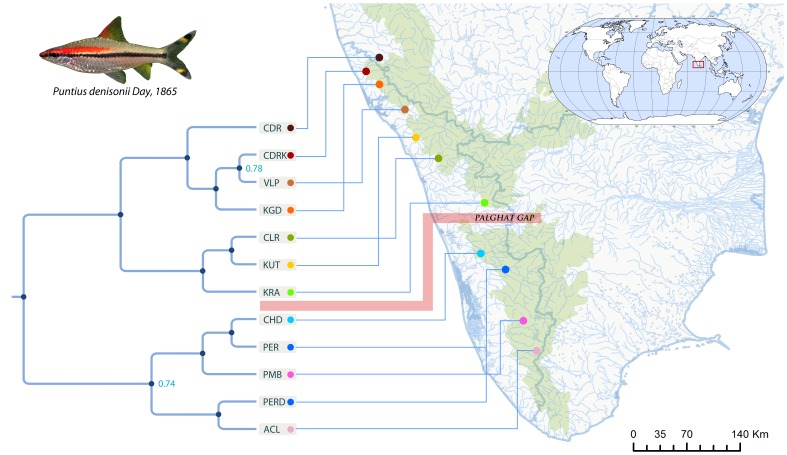
Map showing distribution range of RLTBs and rivers from where samples were collected. Map showing distribution range of RLTBs and rivers from where samples were collected, the species-tree built in *BEAST is shown on the left side. Posterior probability values below 1 are shown at the nodes. Photograph of a specimen considered as *Puntius denisonii* is shown; notice the absence of a black spot on the dorsal fin which is the current diagnostic character for distinguishing it from its congener *Puntius chalakkudiensis* found at location CHD in the map, the tip label codes are explained in Table 1. Note that according to the current taxonomy of RLTBs *P. denisonii* (most probably population PER-D), is nested within the different populations of *P. chalakkudiensis,* the other populations of *P.denisonii* are distributed above the palaghat gap. Here we show that each of these populations (labeled) qualify as evolutionarily distinct lineages.

The endemic red lined torpedo barbs (RLTBs) are represented by *Puntius denisonii* an extremely popular aquarium species and its sibling *P. chalakkudiensis*, significant numbers of which are being collected from the wild [25], [26]. RLTBs occur as fragmented populations (figure 1; Table 1) in 14 small rivers in the WG [27], [28]. However, due to their restricted distribution, unregulated exploitation, decrement in habitat quality and population decline, both species are currently listed as Endangered in the IUCN Red List of Threatened Species [27], [28]. In spite of their public appeal, popularity and conservation importance, the RLTBs have, however, received little scientific attention. Uncertainty still exist on whether the RLTBs comprise one [29], [30], two [31], [32] or more species [12].

**Table 1:** Micro-level distribution of the eight evolutionarily distinct lineages (EDL) including the two recognized species of RLTBs in the Western Ghats.

Lineage	Distribution	Remarks
**CDR**	Tributaries of Chandragiri River in Karnataka part of WG	Northern most distribution range of RLTBs; an EDL
**CDRK, KGD, VLP**	Tributaries of Chandragiri River in Kerala part of WG;Karyangode and Valapattanam Rivers	An EDL
**KUT, CLR**	Kuttyadi and Chaliyar Rivers	An EDL
**KRA**	Bharatapuzha River	An EDL
CHD, PER	Chalakudy and Periyar Rivers	Type locality of the currently recognized species *Puntius chalakkudiensis* is in River Chalakudy [88]
PERD	Periyar River^1^	Occurs in sympatry with *Puntius chalakkudiensis*; an EDL
PMB	Pampa River^1^	An EDL
ACL	Achankovil River^1^	Southern most distribution range; An EDL

1 The precise type locality of *P. denisonii* is still unclear. Three river systems, Periyar, Pampa and Achankovil drain the larger landscape in and around from where Francis Day described *P. denisonii* [89].

Lineages in bold represent the most heavily collected locations of RLTBs, see also figure S8. Voucher specimen of the specimens examined in our study are currently deposited at the museum of the Conservation Research Group (CRG), Department of.

Aquaculture, St. Albert’s College, Kochi, India.

Here, we tested whether the RLTB populations, as a result of geographic isolation and their distribution in isolated (not interconnected) rivers, could be considered as distinct lineages. Our analysis uncover eight evolutionarily distinct lineages that advance our understanding of cyprinid evolution in the WG of India, but at the same time raising numerous conservation and management challenges for one of the world’s most popular freshwater aquarium species.

## Results

### Morphological Analyses

**Figure 2:**
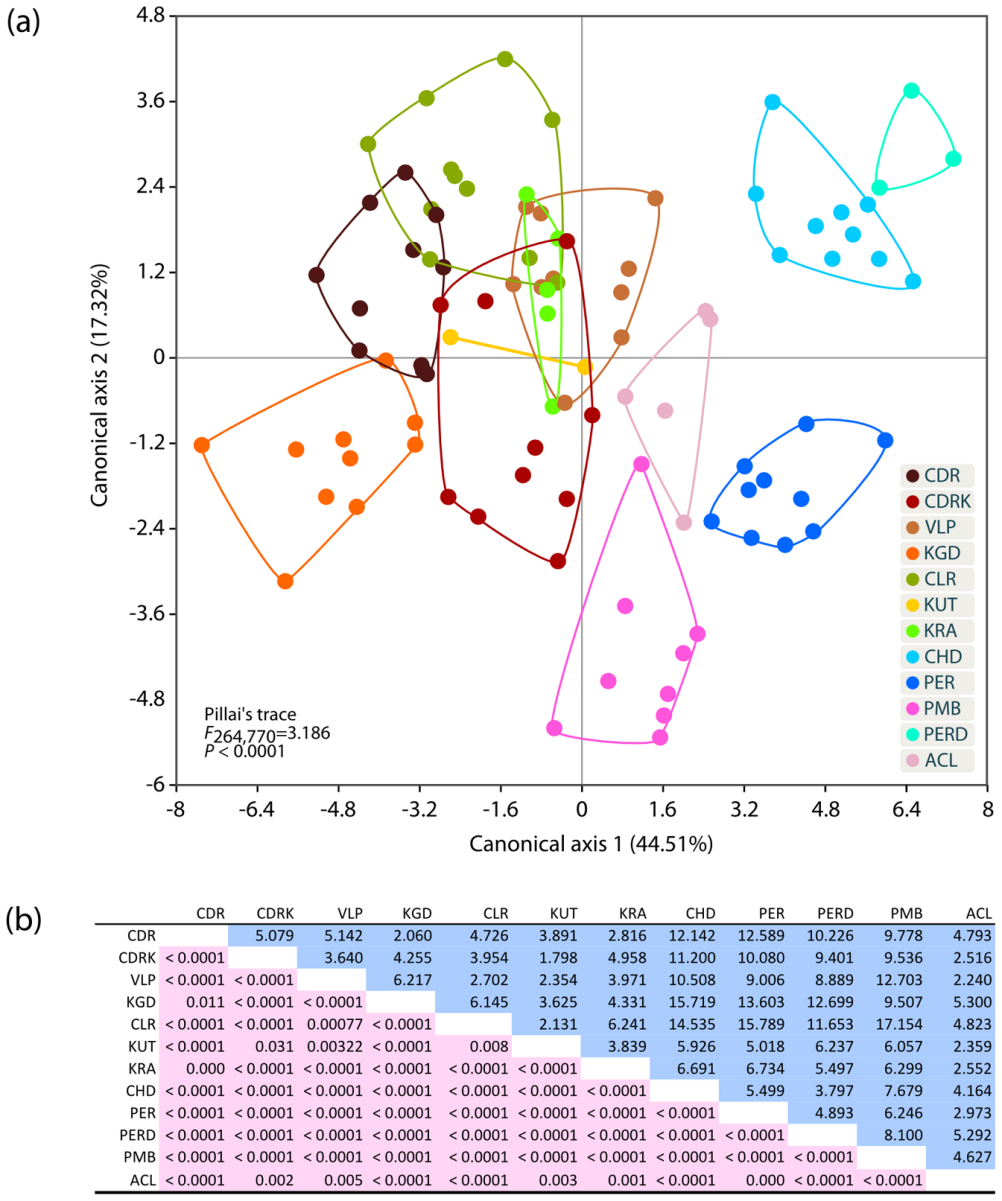
MANOVA/CVA discriminating different RLTB populations. MANOVA/CVA of 24 size adjusted biometric characters of 12 RLTB populations. (a) Clusters of all 12 populations on the first two canonical axis and (b) pair wise matrix of Fisher’s distances between the centroids of the clusters (upper diagonal) and P values for Fisher’s distances (lower diagonal). Percent discrimination by each canonical axis is shown in parenthesis.

Univariate analysis of normality suggested that 24 out of 28 characters were normally distributed. After removing these four variables the resultant matrix of 24 characters did not deviate significantly from multivariate normality (Doornik and Hansen [33] omnibus, Ep = 56.68, P = 0.1829). All size-adjusted characters were significantly different for the 12 studied populations (Table S1). MANOVA/CVA [34] extracted 11 factors out of which the first two axes explain 61.63% of the total variation. The null hypothesis that the mean vectors of the 12 groups are equal was rejected (Pillai's trace = 6.361, F_308,726_ = 3.232, P<0.0001) and Fisher’s distances between the groups suggested that all 12 populations formed significantly different clusters (figures 2 and S2). Based on the distribution of the populations along the first canonical axis, the 12 populations formed two feeble clades (figure 2a), one comprising the populations north (CDR, CDRK, VLP, KGD, CLR, KUT and KRA) and the other south (CHD, PER, PERD, PMB and ACL) of the Palghat (or Palakkad) gap, a major geographical discontinuity in the WG at 11°N (see [4]). Among the multiple variables separating the northern populations from the southern ones (Table S2), the two most prominent characters were comparatively greater head length and smaller caudal peduncle depth in the northern populations. This distinction of two separate clades of northern and southern populations was also supported by non-metric multidimensional scaling (NMDS) of the centroids where the southern populations were distributed along the negative axis while northern populations were distributed along the positive axis of the first NMDS axis (figure S1). Species discrimination in different RLTB populations could therefore only be resolved with complex linear discriminant functions (Table S3), and not by univariate comparison between populations (figure S3). Removing the four non-normally distributed characters from the parametric analysis did not substantially influence the statistical analysis, and NPMANOVA performed on all 28 size adjusted variables suggested that our results were qualitatively similar with significant difference among 12 populations (number of permutations = 100000, F = 7.999, P = 0.00001).

### Genetic Analyses

The initial evaluation of our data suggested good phylogenetic signal as evidenced by having more than 90% of the quartets resolved in the likelihood mapping procedure for both the alignments (see materials and methods and figure S6). The species-tree from *BEAST [35] identified each of the 12 *a priori* designated groups as distinct clusters with posterior probability of 1.0 (figure 1), (only one terminal split (CDRK-VLP) (figure 1) had a posterior probability of 0.70). However, an initial maximum likelihood phylogenetic tree constructed using the concatenated alignment showed only eight distinct clades (figure S4).

**Figure 3:**
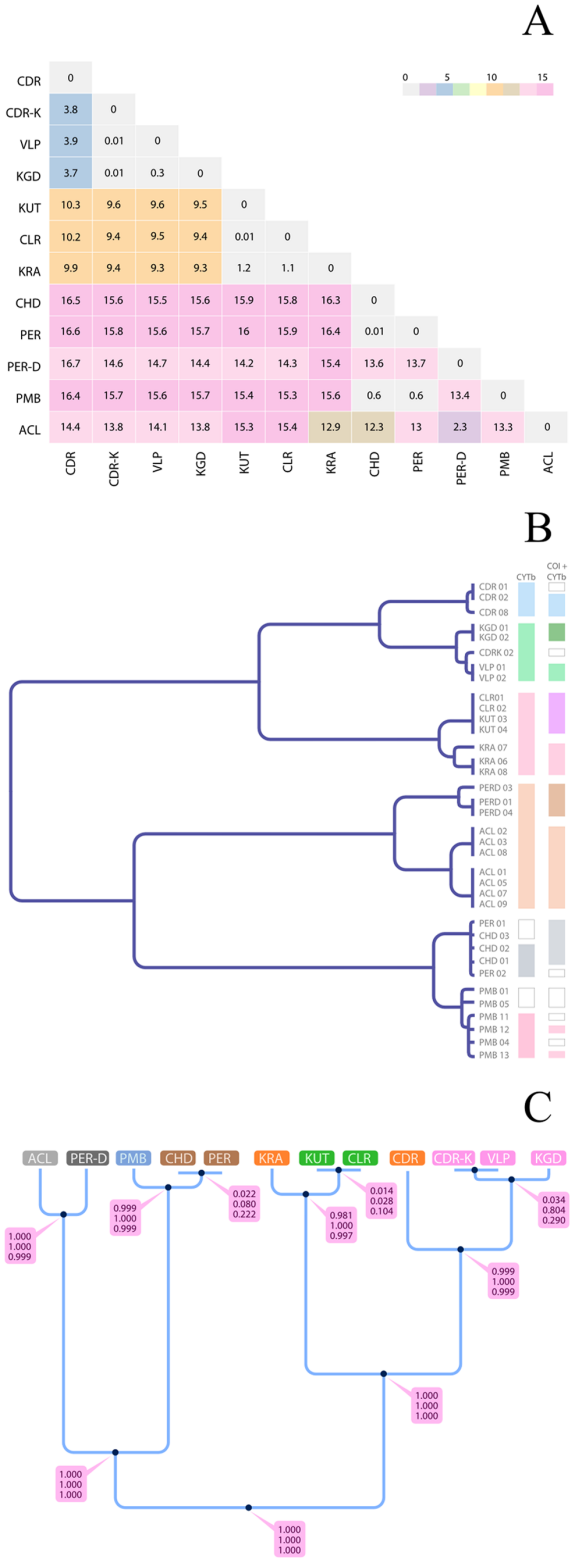
Results of the DNA based species delimitation methods. Results of the DNA based species delimitation methods a) Heat-map showing the fixed distance threshold method and the clustering of the specimens; b) results of the GMYC method implemented on the cytb and cox1+ cytb ultrametric trees, the coloured blocks on the right side indicates the tips clustered together by the program as a same (putative) species; c) Result of the Bayesian method applied on the RLTB species tree, values at nodes are the speciation probabilities using three different prior settings (see materials and methods for details); each evolutionarily distinct lineage (tip) is denoted with a distinct colour.

Seven evolutionarily distinct lineages (figure 3a) were discriminated by a fixed distance threshold of ≥1% used as a standard distance to discriminate species by the BOLD systems of the DNA barcoding consortium [36]. GMYC method [37] suggested that the single threshold model was a better fit to the data than the null model (LRT = 9.03×10^−10 ^for *cytb* tree, and 4.78×10^−7 ^for concatenated tree). Similarly the multiple threshold model fit the data better than the null model (LRT = 3×10^−3^ for *cytb* and 9.84×10^−7^ for concatenated tree). The multiple threshold model distinguished six lineages based on the *cytb* tree and nine lineages based on the concatenated ultrametric tree (figure 3b; see also table S4).

When assuming 12 populations (tips), based on a guide tree produced using *BEAST [35], bayesian species delimitation (bpp) [38] supported eight distinct lineages with posterior probabilities of >0.98 on 7 out of the 11 nodes on the guide tree (figure 1). Different prior distributions on the ancestral population size (θ) and root age (τ) did not affect these results (figure 3c). Thus, the multiple-lineage model explained the data better than the single lineage model as evidenced by the higher posterior probabilities for a multiple species-tree and high (>0.98) speciation probabilities on the nodes of the guide tree.

In short, the Bayesian coalescent analysis and the ML tree identified eight lineages with high probability. The multivariate analysis (based on morphology) and the GMYC methods (based on concatenated dataset) support those eight clades, and also identify others in addition, while the fixed distance threshold methods support seven out of the eight clades identified by Bayesian and the ML methods. Thus, by integrating both morphological and molecular results we propose that RLTBs consists of at least eight evolutionarily distinct lineages, i.e., the number of distinct populations identified with high probabilities and corroborated by both morphological and molecular methods (see Table 1).

### Divergence Time Analysis

**Figure 4:**
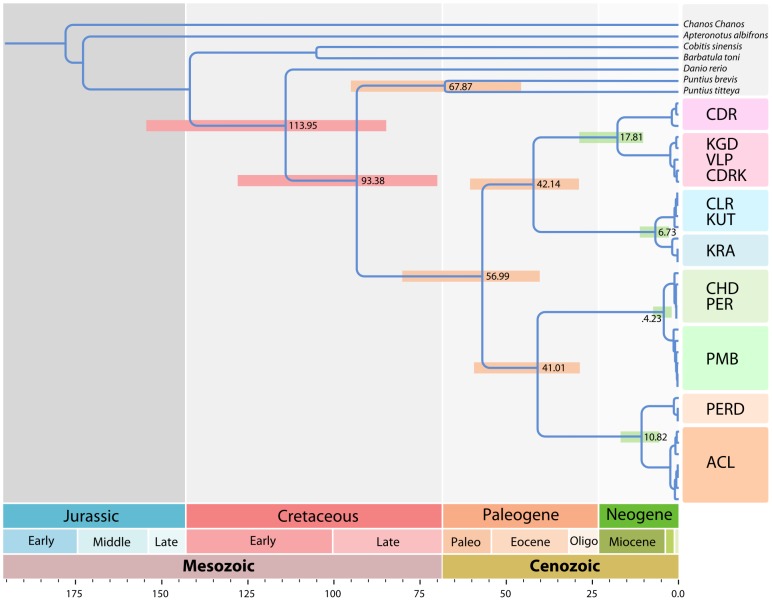
Results of Divergence time analysis. Timetree showing the divergence times of the major RLTB lineages, node bars denote the 95% credibility interval; values at nodes indicate the mean age in million years.

We employed fossil calibrations ([39]–[42]; see materials and methods for details]), and constraints to estimate the divergence times of the RLTB populations. The ancestor of the RLTBs was estimated to have given rise to two lineages around 59 Ma on north and south of the Palghat gap. Further splits around 28–40 Ma in the Eocene due to vicariance of the lineages from two ancestral stocks eventually gave rise to eight evolutionarily distinct lineages at around 5 Ma in its present distribution pattern (figures 1 and 4; tables 1 and S5). An extended divergence time dating analysis by adding the sequences generated in this study with an earlier (larger; cypriniform) dataset [43], showed that the dates (ranges) that we recovered with the MCMC analysis with a smaller dataset are corroborated by the dates recovered from the analysis of the larger dataset (figure S7).

Finally to demonstrate that the RLTB EDLs identified here could be managed as distinct conservation targets, we used phylogenetic methods and identified that sequences hypothesized as to belong to distinct lineages earlier [12] in fact belongs to different EDLs identified in this study. Specimen identified as *Puntius denisonii* and deposited to NCBI belonged to distinct RLTB lineages - CDR (JF915637) and VLP-KGD (JF915638) (figure S8).

## Discussion

Using morphological and various DNA based delimitation methods we provide significant new knowledge on population differentiation of RLTBs. Morphometric analysis and the initial species tree suggested that all 12 populations were distinct. However, the Bayesian coalescent method (and the ML tree) supported only eight lineages with high posterior probabilities (also corroborated by multivariate methods and the GMYC method based on concatenated data), which could signal to a scenario where some populations, even though geographically separated into different river systems, have not genetically diverged significantly. Thus, conservatively we have considered RLTBs to be composed of eight evolutionarily distinct lineages (Table 1). Our study also validates preliminary claims on cryptic diversity within the RLTBs (e.g. [12]).

Morphometric analysis delineated all allopatric populations of RLTBs as distinct. However, it should be noted that the morphological variation observed during the detailed (multivariate) examination are not simple cladistic characters that can be used in the field. Despite the fact that the populations formed different clusters, a univariate analysis of the different size adjusted parameters (figure S3) could not extract distinct character(s) to separate any one population from the rest. However, multivariate discriminant functions (Table S3) could identify an individual belonging to each population, in all cases tested, except in one case where an individual of CDRK population was assigned to CLR in the confusion matrix (Table S6). This illustrates the complexity in discriminating cryptic populations using morphological analysis indicating that morphological segregation among/between populations can be understood only by a combination of characters.

The Palghat gap has been suggested as a biogeographic barrier [5], which has separated species and/or genetic lineages of several taxa including plants [44], amphibians [45], [46], birds [47] and elephant [48]. Our findings support previous studies and indicate that this biogeographic barrier might have played an important role in the distribution of freshwater fishes. Interestingly, the morphological analysis (figures 2a, S1 and S2) also suggests that the RLTB populations south of the gap have diverged from each other more than those found north of the gap.

The divergence times obtained in our study are largely concordant with earlier results. An earlier study with the complete mitochondrial genome sequences of 56 cypriniform fishes estimated the origin of Cyprinidae as 124 Ma ([107.2–143.5 Ma interval; see [41]), while in our analysis the mean age for the emergence of Cyprinidae is ∼114 Ma. The same study [41] obtained a mean age of 155 Ma for basal Cypriniformes, while in our analysis we recovered an age of ∼142 Ma (109–191 Ma interval). However, it should be noted that in a recent study comparing the mitochondrial and nuclear gene based divergence times for fishes [49], Cypriniformes had a mean age of 94.5 Ma (79–113 Ma interval). The same study also showed that mitochondrial based divergence time estimates are significantly higher than nuclear gene based divergence time estimates. However, both the above cited studies recovered somewhat similar ages at the base of Ostariophysi (239 Ma using mitochondrial dataset [41] and 227.8 Ma using nuclear dataset [49]. We suggest that the divergence time estimates that we present should only be considered as a preliminary hypothesis and the RLTB as well as inter-cypriniform divergence times should be validated with larger nuclear and mitochondrial gene datasets. Finally, we generated divergence times for the RLTBs using the sequences from this study in conjunction with a larger cypriniform dataset published earlier [43]. The ages that we recovered for our dataset using the MCMC analysis [50] were corroborated by the dates obtained from the larger dataset using a penalised likelihood method [51].

Further, the divergence time analysis provided evidence that all RLTB populations were separated more than 5 Ma (figure 4). An argument we place is that most of the evolutionary significant lineages identified in this study (except one pair of PER vs. PERD) were products of riverine isolation/separation (vicariance) events around ∼5 Ma that precluded gene flow among these populations. Allopatric speciation is often observed in populations inhabiting geographically isolated areas with similar ecological characteristics and those events are mostly non-adaptive (as opposed to adaptive radiations), where accelerated evolution of traits and phenotypic divergence are typically absent [52], [53]. Our analysis provides the first evidence for population segregation and cryptic diversity among the different isolated populations of RLTBs, which should be further validated with wider sampling and an extended molecular marker dataset (e.g., both mitochondrial and nuclear DNA loci).

While our morphological and species-tree methods differentiated all the 12 allopatric RLTB populations, we used the coalescence-based methods in addition to the fixed distance threshold method to add confidence and determine the exact number of evolutionarily distinct lineages.

The fixed distance threshold based method could separate the various RLTB populations into seven evolutionarily distinct lineages (figure 3a) when a 1% distance threshold was applied [36]. The GMYC model [37], [54] supported the six EDLs based on the *cytb* ultrametric tree and nine EDLs based on the concatenated dataset's ultrametric tree (figure 3b). According to GMYC (on the concatenated tree) VLP and KGD are distinct lineages, however the distance based method and the bayesian methods failed to observe such a case. The failure of GMYC methods to observe the same number of EDLs based on different trees (*cytb* and concatenated) could be due to the fact that this method is sensitive to sample size [55]. Analysis of our species-tree with three different combinations of priors for population size and divergence time in *bpp* identified eight EDLs (figure 3c). The GMYC method applied to the concatenated dataset (ultrametric tree) supported all the eight distinct lineages identified by Bayesian method while the fixed distance threshold method (based on the concatenated dataset) identified seven out of the eight EDLs identified by Bayesian method.

The DNA based methods employed here are not without caveats. DNA barcoding and fixed distance metric as a tool for species delimitation has been a source of contention among various researchers [56]–[59]. GMYC method is known to suffer from phylogenetic inconsistency, rapid evolution in lineages, differences in effective population sizes and discordance among the phylogenetic signal of the loci [60]. The Bayesian method employed here is also known to produce false positives when the sample sizes are less [61]. We overcome these caveats by applying the DNA based methods in conjunction with morphological methods to guide against producing false positives (splitting populations as distinct).

Our study also highlights that fixed distance based methods could give sub-optimal (false negatives) results when used for delineating cryptic species (with recent divergence times) such as the ones in the present study. In addition species delimitation based on evolutionary theory and those statistically testing alternating hypotheses regarding speciation are theoretically better than simple distance based thresholds [62]. We also highlight the problem of phylogenetic uncertainty giving erroneous results with GMYC by showing that two different trees showed different results. GMYC method also failed to classify some tips, which should be mostly due to the low sample size [62]. Thus, larger sample sizes and a robust ultrametric tree are essential for the model to recover correct results. However, our choice to use three different DNA based methods, and more importantly two different coalescent methods, proves imperative since we could choose only the results that are concordant in different methods.

While the morphological data analysis differentiated each population as distinct, DNA based methods could identify only eight distinct evolutionary lineages with high confidence (*bpp* and ML tree; figures 3c and S4). Thus, we propose that the different isolated populations of RLTBs consist of a minimum of eight differentiated lineages, the minimum number of lineages agreed by coalescent and morphological methods, which should receive separate conservation attention and be considered as eight distinct management units.

Finally, we demonstrate the efficacy of managing RLTBs as distinct management units by applying molecular methods to identify the EDL which a sequence (or specimen) of RLTB belongs to, by using two sequences from an earlier publication on aquarium trade [12]. The study [12] had also hypothesized the presence of multiple lineages within RLTBs using the data from these two sequences. Here we constructed a phylogenetic tree (figure S8), and demonstrate that those two sequences belong to different EDLs identified here JF915637 to CDR and JF915638 to VLP-KGD.

Studies with small sample sizes like the present one are inevitable, when dealing with endangered species with populations distributed even inside protected areas. Future use of multilocus nuclear markers with an increased sample size and the application of coalescence-based methods [63] should yield confidence to the present results. Moreover, detailed taxonomic studies should validate the species status of the evolutionarily distinct lineages recognized in this study. Distinguishable morphological characters are essential to discriminate species with easiness in field. Such a morphological character key in conjunction with genetic evidence is the prerequisite to distinguish RLTB populations as distinct species in an integrative taxonomic approach [63]–[66].

Although the coalescence-based techniques used here have been useful for species delimitation including description of new species (see [63] and the references therein), there have been concerns on the use of mtDNA (and DNA sequences) for such purposes [64]–[66]. We have overcome such problems by using different DNA based methods, with an mtDNA dataset (figure S6) that had enough phylogenetic signal [67], in conjunction with morphological methods. Furthermore, we have been cautious in not overemphasizing our results, and suggest that while the discrete populations identified here could indeed be distinct species, they should at present be only considered as ‘Evolutionary Significant Units’ [65].

### Conclusion

Using the popular RLTBs as a case study, we unravel unrecognized diversity among poorly known yet threatened tropical endemic freshwater fish species. Coalescence-based methods led us to discover eight evolutionarily distinct lineages among the isolated RLTB populations. While the advantages and limitations of coalescent-based methods have been discussed recently [63], this method can be extremely useful to supplement biodiversity and taxonomic investigations, and facilitate conservation planning in tropical regions facing the taxonomic impediment. Collecting multilocus datasets could, nevertheless, be prohibitively expensive and, turn away researchers in resource poor (developing and under-developed) nations from using such methods [63]. However, our study demonstrates that even with low sample sizes and few loci, this technique can be adopted by researchers with minimum resources, provided they are used in conjunction with morphological data and with a wide range of samples. Overall, this study advances our understanding of diversity and distribution of freshwater fishes, which comprise one of the world’s most threatened vertebrate groups.

Our findings of the unrecognized diversity in the RLTBs in the form of evolutionarily distinct lineages have considerable impacts for conservation at both local and global scales. Millions of RLTBs are collected (from wild) and exported from the WG since the 1990s (see [26]). Conservation plans, such as ranching, stock enhancement, translocations and reintroductions, require the ability to distinguish populations, and their evolutionary and ecological boundaries [67]. Our study provides the required information for planning and executing such strategies. The conservation/management units identified in this study can also form the basis for future Red List assessments for *P. denisonii* and *P. chalakkudiensis* [27], [28].

## Materials and Methods

A dataset of two mitochondrial gene sequences (*cox*1 and *cytb*) and 28 morphometric characters of RLTBs collected from ten rivers throughout its distribution range (figure 1, Table 1) was generated. The molecular dataset consisted of an average of 2.9 individuals per population and the morphological data consisted of an average of 7.9 individuals per population.

### Ethics Statement

Specimens were procured from aquarium collectors and/or directly collected from the wild. Permits for collection of fish inside Protected Areas (PA's) were provided by Kerala State Forest and Wildlife Department (No.WL12-8550/2009) (applicable to four of the sampled sites: ACL, PER/PERD, PMB, CHD; see figure 1). Two of the sites from where fishes were collected (KRA and KUT; figure 1) fell outside PA's and therefore no permits were required. From the remaining sites, fishes were procured from local aquarium collectors. Fishes were captured by backpack electro-shocker (in sites from where we collected directly) and eco-friendly seine and bag nets (in case of material collected by aquarium fish collectors). For downstream molecular biology protocols, a small piece of tissue from the lower lobe of the caudal fin (fin clip) was excised, and subsequently (whenever possible) the fishes were released back into the same habitat. For morphometric analyses, fishes were transferred to ice slurry post anaesthetization (in 200 mg/L Tricaine Methane Sulphonate (MS222)) and transported to the laboratory. We chose ice slurry because the fish had to be transported to long distances (in some cases ∼300 kms) without compromising on the morphological characters (shape, color) that are essential for taxonomic investigations. Details of samples used for the study is provided in Table S7. Institutional ethics committee of St. Albert's College, Kochi, Kerala, India (SAC-IAEC 2005-01) approved the design and implementation of the study.

### Morphological Measurements and Analysis

Measurements were made point to point with dial calipers to the nearest 0.1 mm. Counts and measurements were made as far as possible on the left side of specimens following standard methods for cyprinid taxonomy [68]. Care was taken to use adult (or large) specimens for the analysis. To nullify the effect of size, size adjusted measurements were obtained by expressing subunits of body as percent of standard length (SL) and subunits of head as percent of head length (HL). This size correction assumes that growth of different characters is isometric. Therefore, to check for isometry we plotted log-log plot of each character with the standard length (or head length) and checked whether the slope of the line was significantly different from unity. The slope of the line for all characters was not significantly different from unity as assumed by isometry.

Size adjusted morphometric measurements were used for morphometric analysis of the data. Univariate normality for each variable was checked using Shapiro-Wilk test. Variables that were not normally distributed were removed from further parametric analysis; however, all characters were used in non-parametric analysis. ANOVA was performed to understand whether standardized morphometric characters differed among the populations. Since multiple tests were performed on the same data we applied sequential Bonferroni correction to the α wherever applicable. Multivariate normality of the final data was checked using Doornik and Hansen omnibus [33]. MANOVA (Multivariate Analysis of Variance)/CVA (Canonical Variates Analysis) was performed to check whether the populations form significantly distinct clusters morphometrically [34]. MANOVA/CVA explicitly attempts to model the difference between the groups of data by extracting factors that maximize inter group variation and minimize intra group variations. MANOVA/CVA was chosen as a more appropriate technique than Principle Component Analysis (PCA), which gives equal weight to all the variables and as a result cannot reveal the differences among closely related clusters in less number of dimensions. This is true especially when the groups do not have highly diverged morphological structures. However, since MANOVA/CVA considers prior groups, we tested for intra-group homogeneity by two methods so as to account for the bias created by the grouping method itself. (1) The null hypothesis, which states that the mean vectors of the 12 populations are equal, was tested using Pillai’s trace [69]. (2) We calculated the Mahalanobis distances among the individuals and computed Fisher’s distances between 12 populations (as the distance between the centroids of the two clusters, divided by the sum of their standard deviations) to check if the clusters formed by 12 populations are significantly different. Distances between the centroids of the 12 populations were visualized by performing Non-metric Multidimensional Scaling [70]. To account for any loss of information from the characters, which were not normally distributed, we performed non-Parametric MANOVA (NPMANOVA) [71] on all size adjusted characters to test the null hypothesis that the populations are the same. Statistical analysis was performed in Microsoft EXCEL ®, Systat 12 ® and the freeware PAST [72].

### Genetic Analyses

To yield confidence to the results from the morphometry based analysis, we applied various DNA based delimitation methods, which are described below.

Total genomic DNA from the specimens was isolated using a modified salting out protocol [73]. Partial sequences of two mitochondrial genes, *cytochrome b* (*cytb*) and *cytochrome oxidase* 1 subunit (*cox*1), were amplified using universal primers published earlier [74], [75]. The amplifications were performed in 25 µl reactions containing 1X assay buffer (100 mM Tris, 500 mM KCl, 0.1% gelatin, pH 9.0) with 1.5 mM MgCl2, 10 p moles/µL of primer mix, 10 mM dNTPs), 1.5 U Taq DNA polymerase and 20 ng of template DNA. To evaluate the reliability of the DNA amplification, a negative control was set up by omitting the template DNA from the reaction mixture. The reaction mixture was initially denatured at 95°C for 5 minutes followed by 29 cycles (denaturation at 94°C for 45 seconds, annealing at 50°C (for *cytb*) or 54°C (for *cox*1) for 30 seconds and 72°C for 45 seconds). Reaction was then subjected to a final extension at 72°C for 5 minutes. PCR products were visually inspected for quality and length in a 1% agarose gel and amplicons confirming with the quality checks were subsequently outsourced for sequencing the forward strands.

The DNA sequences were edited using BIOEDIT [76] and translated into amino-acids, confirmed that internal stop codons were absent, aligned using MUSCLE [77], back-translated and used for all downstream analyses. Phylogenetic trees were constructed using maximum-likelihood (ML) method as implemented in TREEFINDER [78]. Before carrying out analyses the phylogenetic signal of the datasets were analyzed using the likelihood-mapping procedure [79]. For the maximum likelihood analysis the best-fit nucleotide substitution models were determined using TREEFINDER [78]. Sequences generated for this study are deposited in Genbank (Table S8). Trace files for each of the sequences are available for download from figshare (http://dx.doi.org/10.6084/m9.figshare.95635).

We carried out three DNA based species delimitation analysis, to identify the evolutionarily distinct lineages: 1) A fixed distance threshold method; 2) general mixed Yule coalescent model (GMYC); 3) bayesian species delimitation as implemented in b*pp v. 2.1a.* For these methods, we built individual gene trees, and also a tree using the concatenated (supermatrix) dataset. In addition to the gene trees we used *BEAST [35] to estimate the species-tree directly from the sequence data, since the bayesian species delimitation method [38] requires a species tree as the input to carry out the analysis. *BEAST incorporates uncertainty associated with gene trees, nucleotide substitution model parameters and the coalescent process [35]. It should be noted that the gene tree contained 35 tips (equal to the number of sequences used), while the species tree contains only 12 tips (equal to the number of populations studied; see figure 3b&c). The GMYC method requires an ultrametric tree, which was generated using the *chronopl* function in *ape v 3.0–4* [51], [80] with a lambda value of 0.01.

DNA barcoding methods use the mitochondrial *cox*1 sequence based fixed distance thresholds to delineate distinct lineages [75], [81]. We calculated the maximum likelihood distances for the concatenated dataset, as opposed to K2P distances (used commonly in *cox*1 based DNA barcoding studies) since it has been shown as inappropriate in most cases (see [36] for details). It should also be noted that we used the concatenated *cox*1*+cytb* dataset not just the *cox*1 sequences for calculating the distances. The dataset was divided into two partitions and distance calculated based on the best-fit nucleotide substitution models HKY+G for *cytb* partition and TVM+G for *cox*1 partition, with five rate categories. Maximum likelihood distance calculation was done in TREEFINDER [78].

The general mixed Yule coalescent model [35], [62] is based on the assumption that there are changes in the branching rates at the species boundaries. The GMYC exploits the predicted difference in branching rate under the two modes of lineage evolution, where the branching patterns within each genetic cluster reflects a neutral coalescent process and the branching patterns between two genetic clusters reflects timing of speciation events, and by assessing the point of highest likelihood of the transition [35], [82] it differentiates the evolutionarily distinct lineages. Monaghan and co workers [54] developed a modified GMYC model that allows for a variable transition from coalescent to speciation among lineages by identifying multiple thresholds reflecting the variable lineage divergence. The likelihood values of the GMYC models are compared to a null model, which assumes a single branching process for the tree, using a Likelihood Ratio Test (LRT).

GMYC clustering was performed using the package *splits v.1.0–11* (SPecies' LImits by Threshold Statistics, http://r-forge.r-project.org/projects/splits/) implemented in R [83]. A maximum likelihood tree using the concatenated dataset and the *cytb* tree (separately) was used to generate the ultrametric tree. We used two different trees *(cytb ultrametric tree and a concatenated ultrametric tree)* with GMYC model to check whether it produced concordant results and recovered the same clades.

*Bayesian Phylogenetics and Phylogeography* software (*bpp v. 2.1a*; [38]) was used to identify distinct evolutionary lineages. This method requires a multi-species multi-gene dataset and also requires that the user assign the candidate groups prior to the analysis, and a phylogeny showing the relationships between the groups. We assumed that each sampling location was a distinct population, since each of the sampling locations are isolated drainages and no gene flow is possible among the populations, except in two cases of PER-PERD and CRD-CDRK. PERD was a morphological variant compared to the commonly occurring specimens PER in river Periyar, while the second group CDR and CDRK occurred in two distant tributaries of River Chandragiri (figure 1, table S1). Thus we had samples from 10 isolated rivers, which we assigned as 12 distinct clusters for the *BEAST and *bpp* analyses.

The MCMC analysis in *BEAST was run twice and a total of 50 million generations (sampling trees every 1000 generations), first 25% trees were discarded as burnin and the convergence was examined TRACER v. 1.4.1 [84]. The species tree was summarised using the *tree-annotator* program from the BEAST package [85] and the tree was visualised and edited using *figtree* (http://tree.bio.ed.ac.uk/software/figtree/).

The Bayesian (*bpp*) method accommodates the species phylogeny as well as lineage sorting due to ancestral polymorphism. This method is based on the assumption of a biological species concept, where gene flow stops at a speciation event [38]. When the user provides a guide tree, which is fully resolved, the program evaluates subtrees by collapsing or splitting nodes (without branch swapping). Under this method, we expect strong support for populations/species isolated for an extended period of time, and weak support for populations/species that have experienced extensive gene flow [7]. The parameters in the model include the species divergence times τ, measured by the expected number of mutations per site, and population size parameters θ = 4Nμ, where N is the effective population size and μ is the mutation rate per site per generation so that θ is the average proportion of different sites between two sequences sampled at random from the population.

The prior distributions on the ancestral population size (θ) and root age (τ) can affect the posterior probabilities for models, with large values for θ and small values for τ favouring conservative models containing fewer species [38]. We evaluated the inﬂuence of these priors by considering three different combinations of prior, similar to an earlier study [7].

The first combination of priors was to set a relatively large ancestral population size θ ∼ G (1, 10) and deep divergence time and τ ∼ G (1, 10) both with a mean of 0.1 and variance of 0.01. The second combination was to set a small ancestral population θ ∼ G (2, 2000) and shallow divergence time τ ∼ G (2, 2000), both with a prior mean 0.001 and variance of 5e-07. The third prior combination set a large ancestral population θ ∼ G (1, 10), with shallow divergence time τ ∼ G (2, 2000). The *rjMCMC algorithm-0* was run with a fine tune parameter of 15 and 20 and was run twice to confirm consistency between runs. The species tree and the sequence alignment used for the analysis are available for download from (http://dx.doi.org/10.6084/m9.figshare.95635). The program outputs the speciation probabilities at each nodes of the maximum posterior probability tree (MAP) tree. A posterior (speciation) probability of >0.95 was considered as a strong evidence of speciation at the node, we also ensured that all the three different priors used produced consistent results.

### Divergence Time Estimation

A phylogenetic tree with *Chanos chanos* (Anotophysi) and *Apteronotus albiforns* (Gymnotiformes) as out-groups was calibrated using fossil ages, with two calibration points as soft bounds: (i) at the base of Cyprinidae; we set a minimum age of 49 Million years (Ma) and maximum age of 59 Ma based on the oldest known cyprinid fossil [39], [40], and (ii) at the base of Ostariophysi, a constraint of 146 Ma was set based on the oldest available ostariophysian fossil [41], [42]. In addition to the fossil calibrations, the root of the tree, at the base of Gymnotiformes, was constrained with a loose upper bound, to a maximum age limit of 239 Ma based on the results of a recent study [41] as the basal time of emergence of Ostariophysi.

We estimated the divergence times at each node of the phylogenetic tree using MCMCtree [50] with a log-normal rate prior and birth-death time prior. Independent rates for each branch was considered, and maximum likelihood estimation of branch lengths was done using HKY85 model [86].

The node at the base of Cyprinidae was based on the oldest cyprinid fossil [39], [40] and was set to 49–59 million years ago. The root node (figure 4) was constrained to an upper bound of 239 million years ago and a lower bound of 146 million years ago [41], [42]. The MCMC algorithm was run for 5×20000 iterations, and first 2000 samples were discarded as burnin. The outgroups for Cypriniformes used for the phylogeny construction and divergence time estimation were *Chanos chanos* (Anotophysi) and *Apteronotus albiforns* (Gymnotiformes). The gamma prior for the overall rate parameter μ was set to G (2,7), with a mean of 0.29 and variance of 0.04. The rates for individual loci were calculated using *baseml* program implemented in PAML package (v.4.4a; [87]), with global clock assumption and fossil calibrations as specified above.

To add confidence to our age estimates, we repeated the dating analysis by integrating our mitochondrial dataset with a larger dataset published earlier [43] and used the same age constraints and carried out divergence time dating with the penalised likelihood algorithm with a rate smoothing value of 0.1 in r8s [51].

### Identifying the RLTB Lineages Represented in Trade

To demonstrate the conservation benefits of managing the RLTBs as distinct management units, we tried to identify which EDLs have been represented in trade using a DNA based approach. We collected two RLTB *cox*1 sequences (JF915638 and JF915637) from NCBI sequenced and published as part of a study on aquarium trade [12]. We produced an alignment (using MUSCLE [77]) of our *cox*1 dataset and these two sequences after translating into proteins, backtranslated the alignment and produced an ML tree with GTR+G+I model and 4 rate classes.

## Supporting Information

Figure S1 Results of non-metric multidimensional scaling. Non-metric multidimensional scaling of DFA functions at the centroid using Euclidian distances. Connecting line is the minimum span tree. Shephard plot is shown in the inset.
(PNG)

Figure S2 MANOVA/CVA on the on the first three canonical axes. (a) Clusters of all 12 populations on the first three canonical axes, (b) clusters of populations north of Palghat gap and (c) clusters of populations south of Palghat gap. Points are connected by line just for eyeballing the clusters.
(JPG)

Figure S3 Box plot of size adjusted morphometric characters. Redline is the mean.
(PNG)

Figure S4 Phylogenetic trees used for the study. Phylogenetic tree constructed using the concatenated alignment showing the relationships between the specimens collected from different river systems throughout their range, shLRT node support are shown, right side of the tree has each group labeled with their river of origin.
(PNG)

Figure S5 Cladogram from the divergence time analysis. Cladogram with corresponding node numbers for which the divergence times are presented in the table S5, tips have their numbers as the prefix followed by an underscore and the specimen name.
(PDF)

Figure S6 Phylogenetic signal of the sequence alignments. Results of the likelihood mapping procedure for the CYTb and COI alignments used in this study, note that more than 90% of the quartets are resolved in both cases.
(PDF)

Figure S7 Results of the extended divergence time analysis. A dataset combining the *cytb* data from this study with the dataset from Ruber et al., (2007) was generated. The date ranges retrieved from our MCMCtree analysis is shown in red and the dates recovered with the extended analysis (with r8s) are shown in pink which are within the ranges of the dates recovered from the analysis of our small dataset.
(PDF)

Figure S8 Tree showing the phylogenetic position of sequences from traded specimen. Tree showing the phylogenetic position of sequences generated for an earlier study [12] on aquarium trade. The sequences belong to two different evolutionarily distinct lineages CHD (PD_JF915637) and VLP-KGD (PD_JF915638), which are known to be the most heavily collected locations for RLTB trade.
(PDF)

Table S1 Analysis of Variance of size adjusted characters.
(PDF)

Table S2 MANOVA/CVA loadings for the first three canonical axes.
(PDF)

Table S3 Discriminant functions for the 12 populations
(PDF)

Table S4 Detailed results of the GMYC methods implemented for the cytb ultrametric tree and the concatenated ultrametric tree, the species distinction made is displayed in the Figure 3b in the main text.
(PDF)

Table S5 Table showing the divergence times for the RLTB's internal node number are in the first column which follows the Figure S5.
(PDF)

Table S6 Confusion matrix for group identity based on discriminant functions. Populations in the row are original identities. Populations in the column are predicted identities. Diagonal elements indicate correct prediction of group identity. Off diagonal elements show wrong predictions.
(PDF)

Table S7 Samples used and sampling sites: a) Samples procured from aquarium collectors, corresponding river systems and number of samples used; b) List of sampling sites from where we collected samples directly, corresponding river systems and number of samples used.
(PDF)

Table S8 Genbank details of the sequences used in the study and codes for the trace files of sequences uploaded at figshare (http://dx.doi.org/10.6084/m9.figshare.95635).
(PDF)
