# Correction: Morphological and Genetic Evidence for Multiple Evolutionary Distinct Lineages in the Endangered and Commercially Exploited Red Lined Torpedo Barbs Endemic to the Western Ghats of India

**DOI:** 10.1371/annotation/bf1622ea-bb3e-49f7-9d16-f549c05fc584

**Published:** 2013-08-30

**Authors:** Lijo John, Siby Philip, Neelesh Dahanukar, Palakkaparambil Hamsa Anvar Ali, Josin Tharian, Rajeev Raghavan, Agostinho Antunes

A recent publication in Journal of Phylogenetics and Evolutionary Biology suggested the possible presence of cryptic diversity and substantial stock differentiation between the two currently described red lined torpedo barb species P. denisonii and P. chalakkudiensis. We have missed this citation in our article, which we now provide below:

John L, Peter R, Gopalakrishnan A (2013) Population structure of Denison's Barb, Puntius denisonii (Pisces: Cyprinidae): a species complex endemic to the Western Ghats of India. Journal of Phylogenetics and Evolutionary Biology 1: 106

Unlike the above mentioned publication, in our paper we employed a wider range sampling and state-of-the-art coalescent based genetic methods and multivariate morphometrics, to reveal that the red lined torpedo barbs comprise a cryptic species complex consisting of eight evolutionarily distinct lineages. 

