# Supplementary figures and images for: Morphological and Genetic Evidence for Multiple Evolutionary Distinct Lineages in the Endangered and Commercially Exploited Red Lined Torpedo Barbs Endemic to the Western Ghats of India
Source: PLoS One. 2013 Jul 22;8(7):e69741. doi: 10.1371/journal.pone.0069741 (PMC3718778; doi:10.1371/journal.pone.0069741)

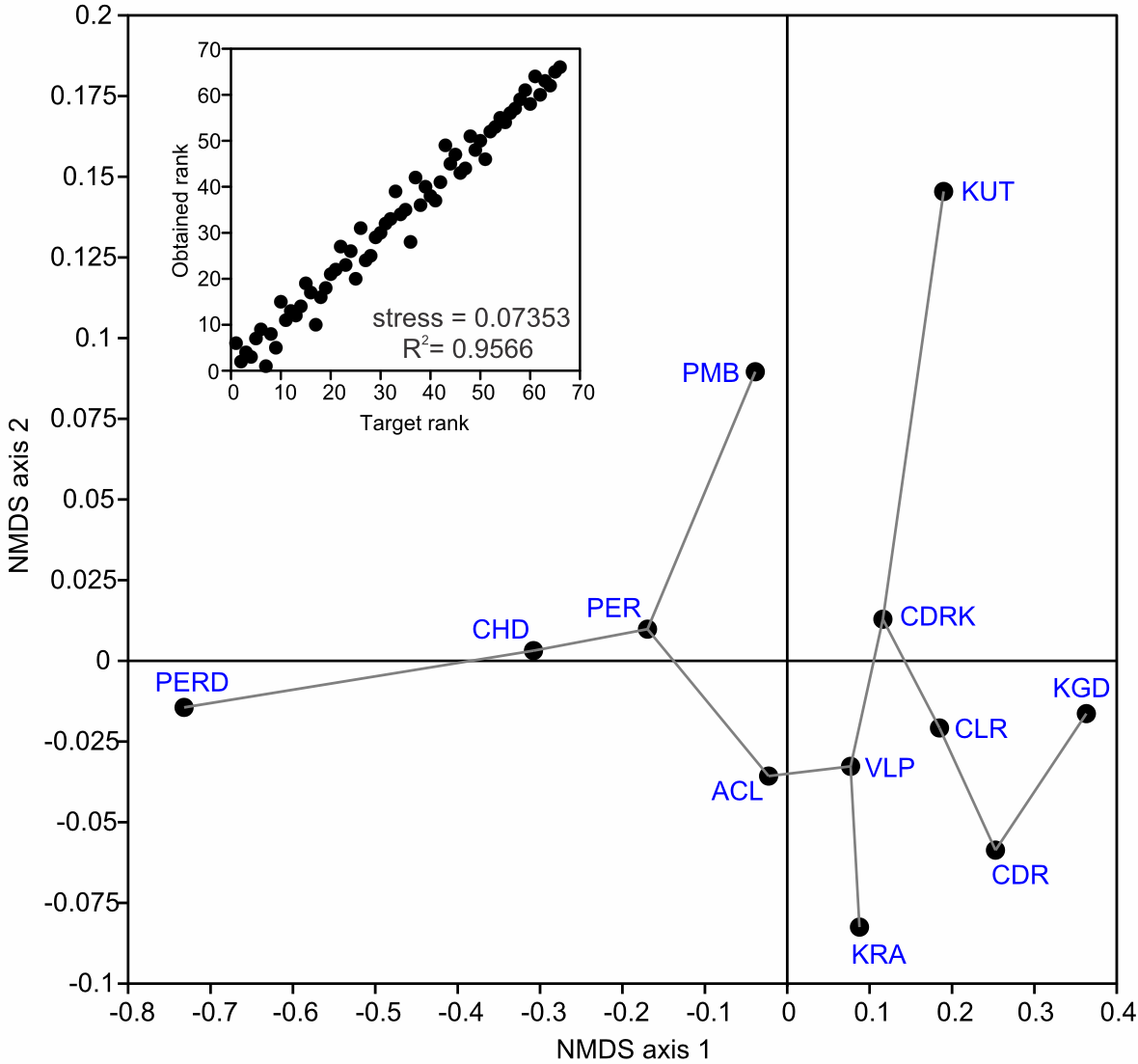

Supplement: Figure S1 — Results of non-metric multidimensional scaling. Non-metric multidimensional scaling of DFA functions at the centroid using Euclidian distances. Connecting line is the minimum span tree. Shephard plot is shown in the inset. (PNG) [file pone.0069741.s001.png]

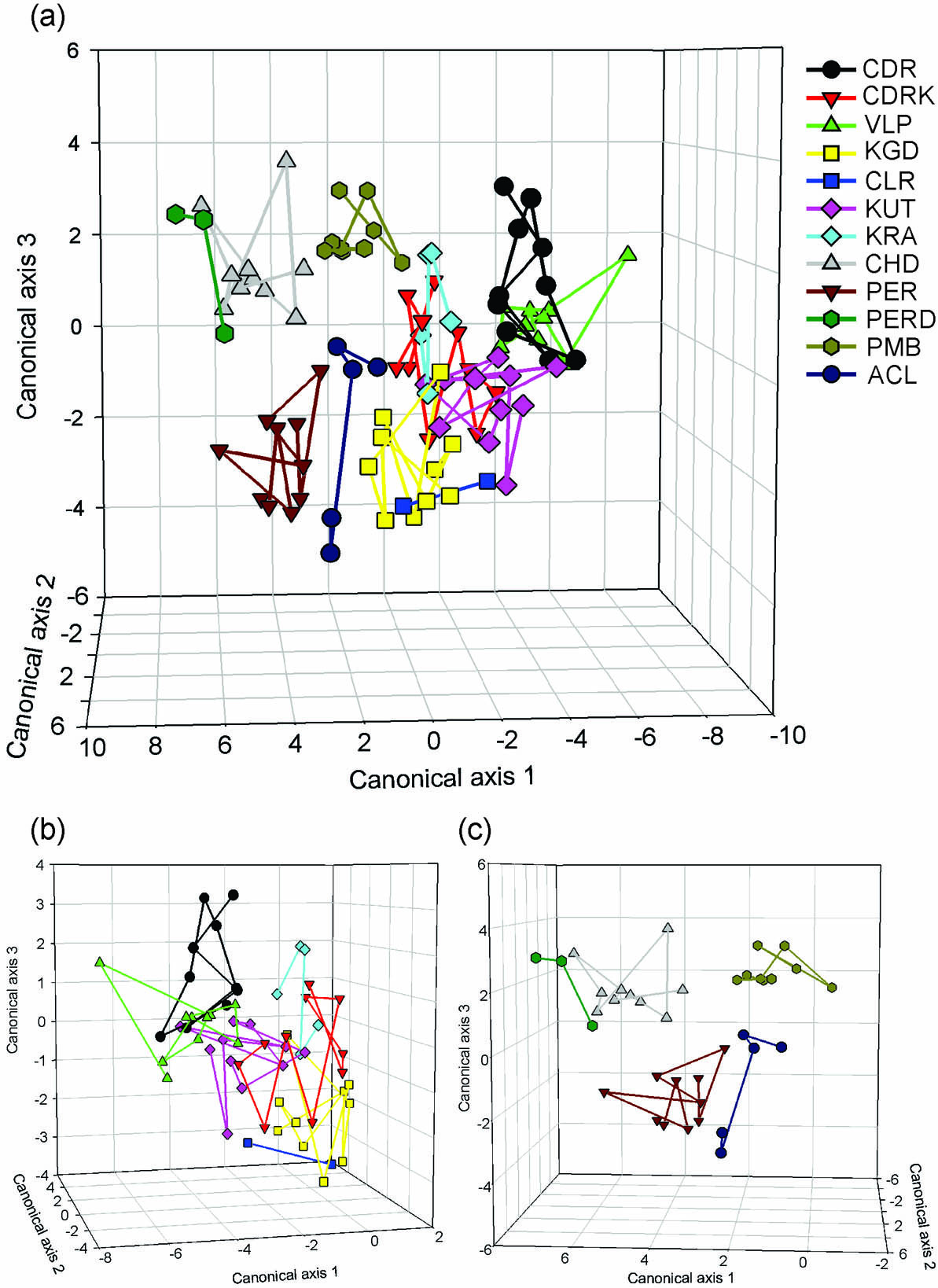

Supplement: Figure S2 — MANOVA/CVA on the on the first three canonical axes. (a) Clusters of all 12 populations on the first three canonical axes, (b) clusters of populations north of Palghat gap and (c) clusters of populations south of Palghat gap. Points are connected by line just for eyeballing the clusters. (JPG) [file pone.0069741.s002.jpg]

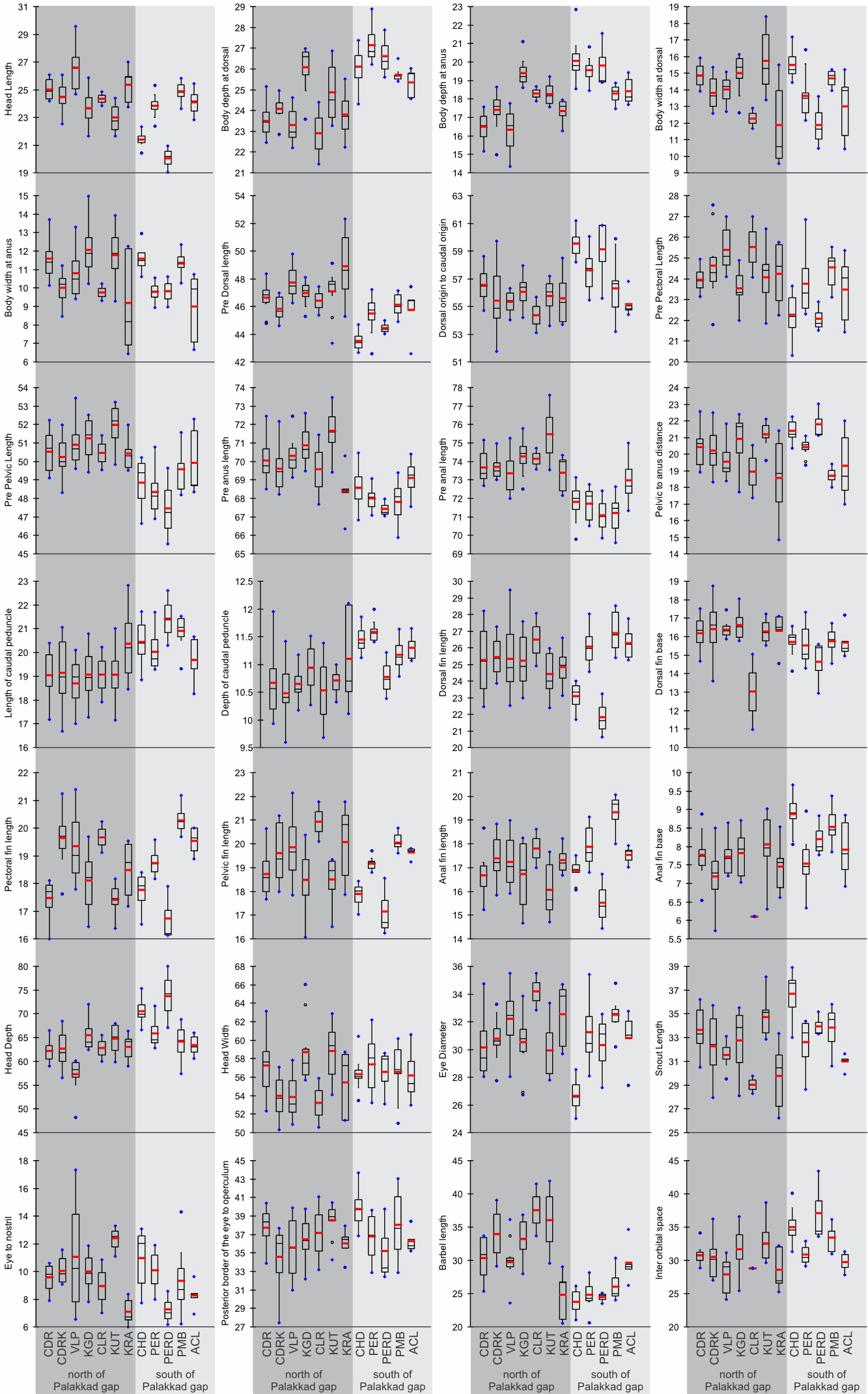

Supplement: Figure S3 — Box plot of size adjusted morphometric characters. Redline is the mean. (PNG) [file pone.0069741.s003.png]

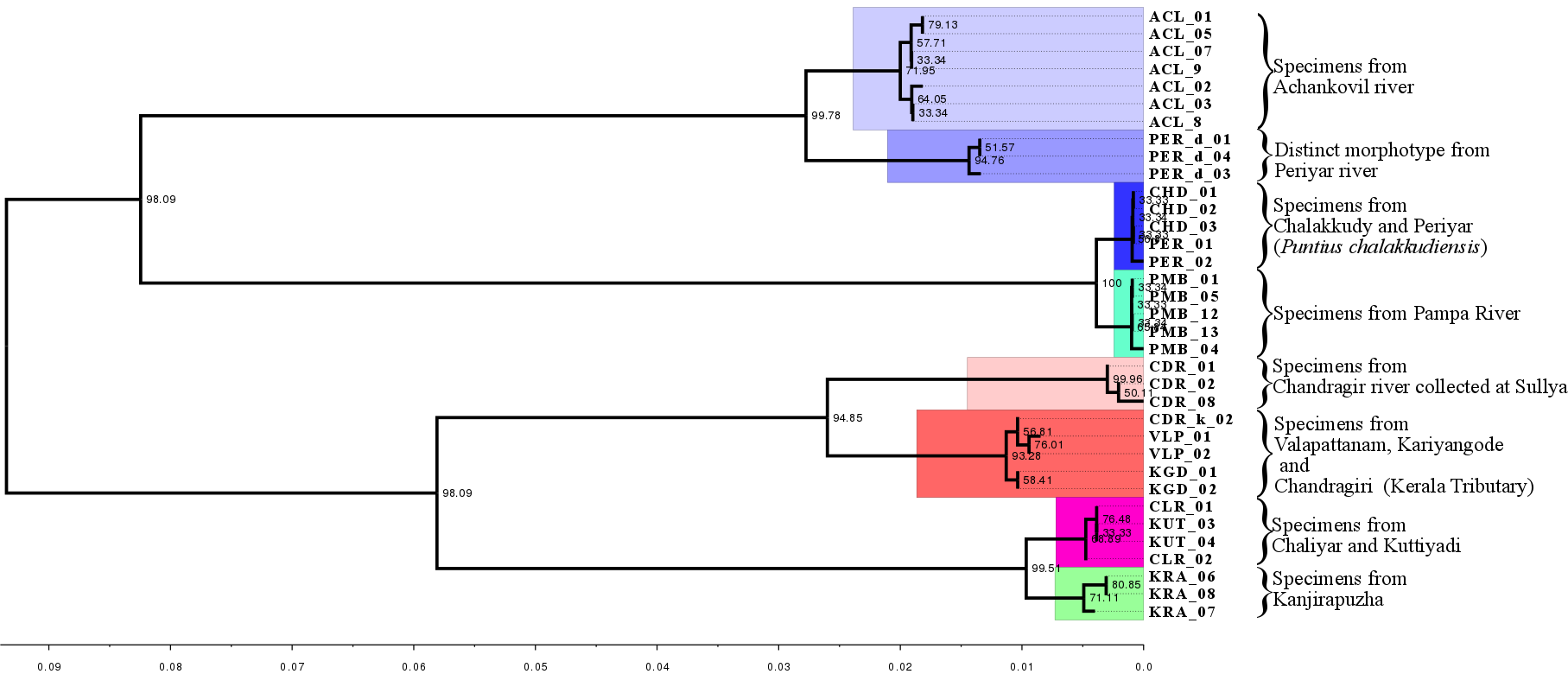

Supplement: Figure S4 — Phylogenetic trees used for the study. Phylogenetic tree constructed using the concatenated alignment showing the relationships between the specimens collected from different river systems throughout their range, shLRT node support are shown, right side of the tree has each group labeled with their river of origin. (PNG) [file pone.0069741.s004.png]

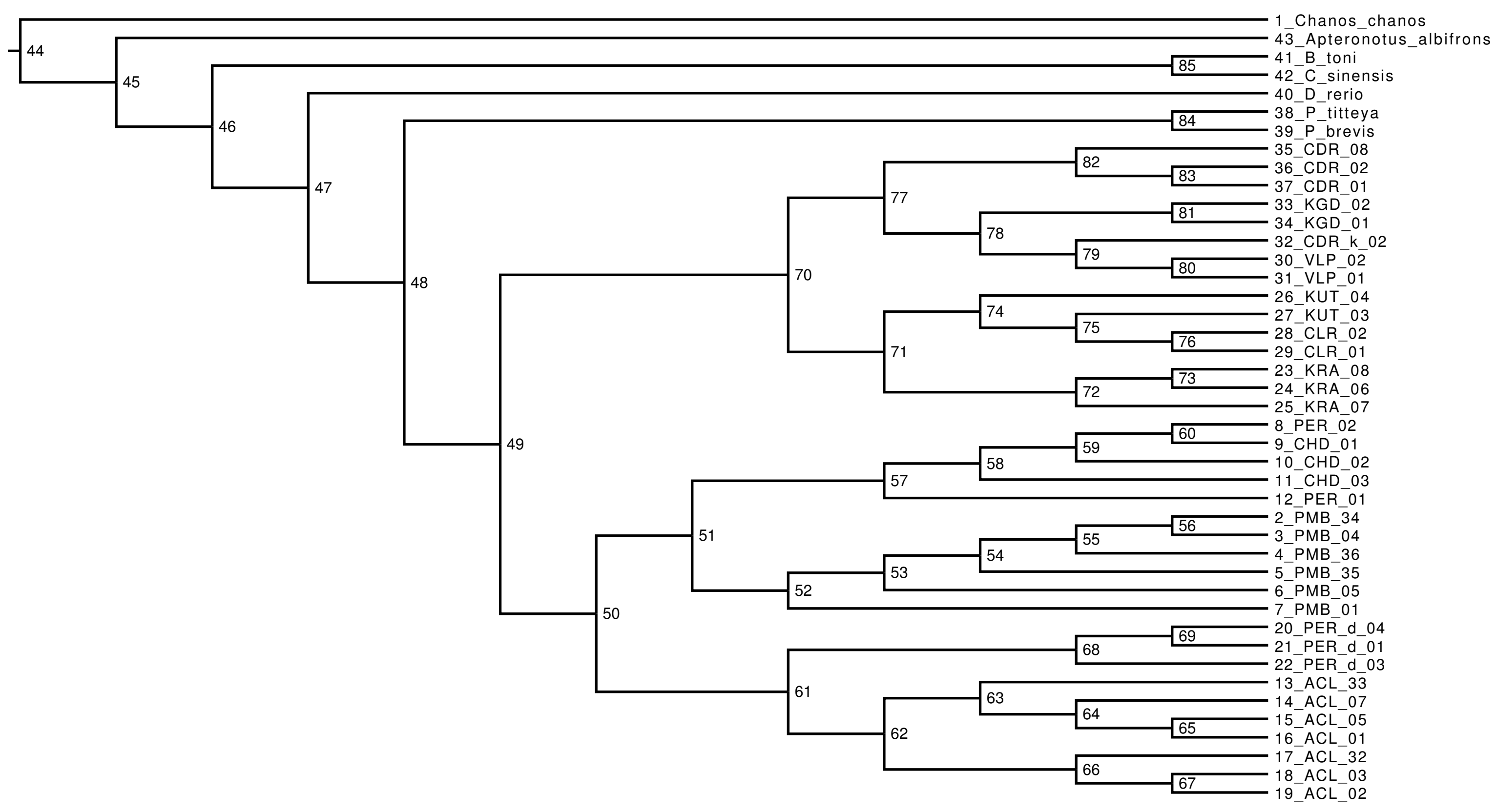

Supplement: Figure S5 — Cladogram from the divergence time analysis. Cladogram with corresponding node numbers for which the divergence times are presented in the table S5, tips have their numbers as the prefix followed by an underscore and the specimen name. (PDF) [file pone.0069741.s005.pdf]

# LIKELIHOOD MAPPING STATISTICS

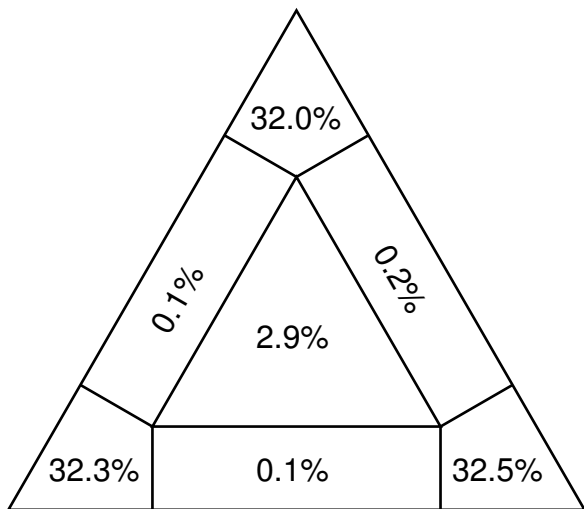

CYTB - RLTB alignment

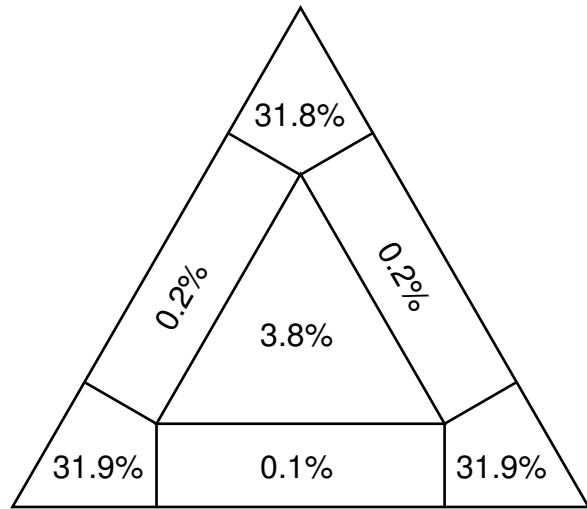

COI -RLTB alignment

Supplement: Figure S6 — Phylogenetic signal of the sequence alignments. Results of the likelihood mapping procedure for the CYTb and COI alignments used in this study, note that more than 90% of the quartets are resolved in both cases. (PDF) [file pone.0069741.s006.pdf]

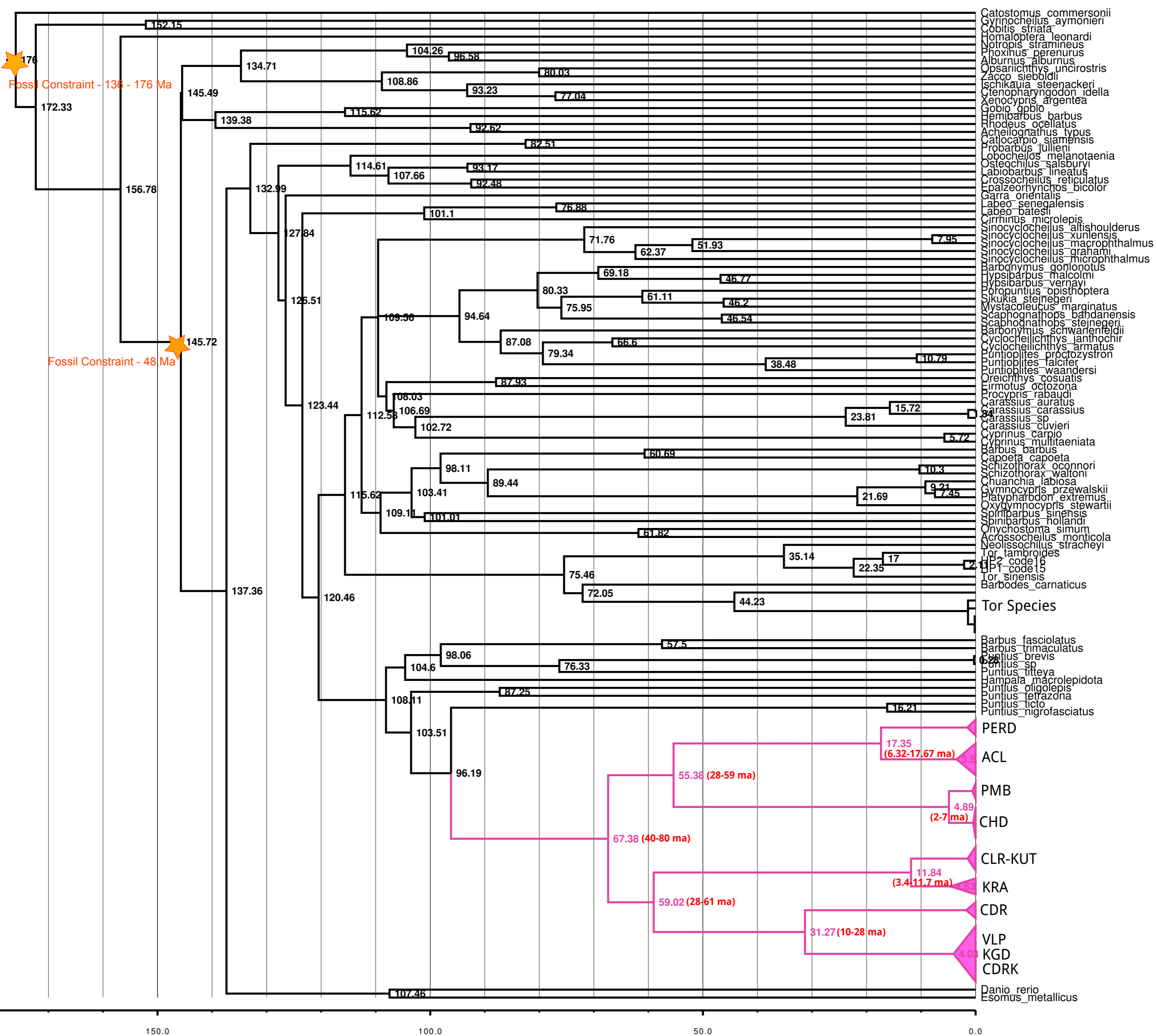

Supplement: Figure S7 — Results of the extended divergence time analysis. A dataset combining the cytb data from this study with the dataset from Ruber et al., (2007) was generated. The date ranges retrieved from our MCMCtree analysis is shown in red and the dates recovered with the extended analysis (with r8s) are shown in pink which are within the ranges of the dates recovered from the analysis of our small dataset. (PDF) [file pone.0069741.s007.pdf]

PhyML ln(L)=-1271.0 687 sites GTR 4 rate classes

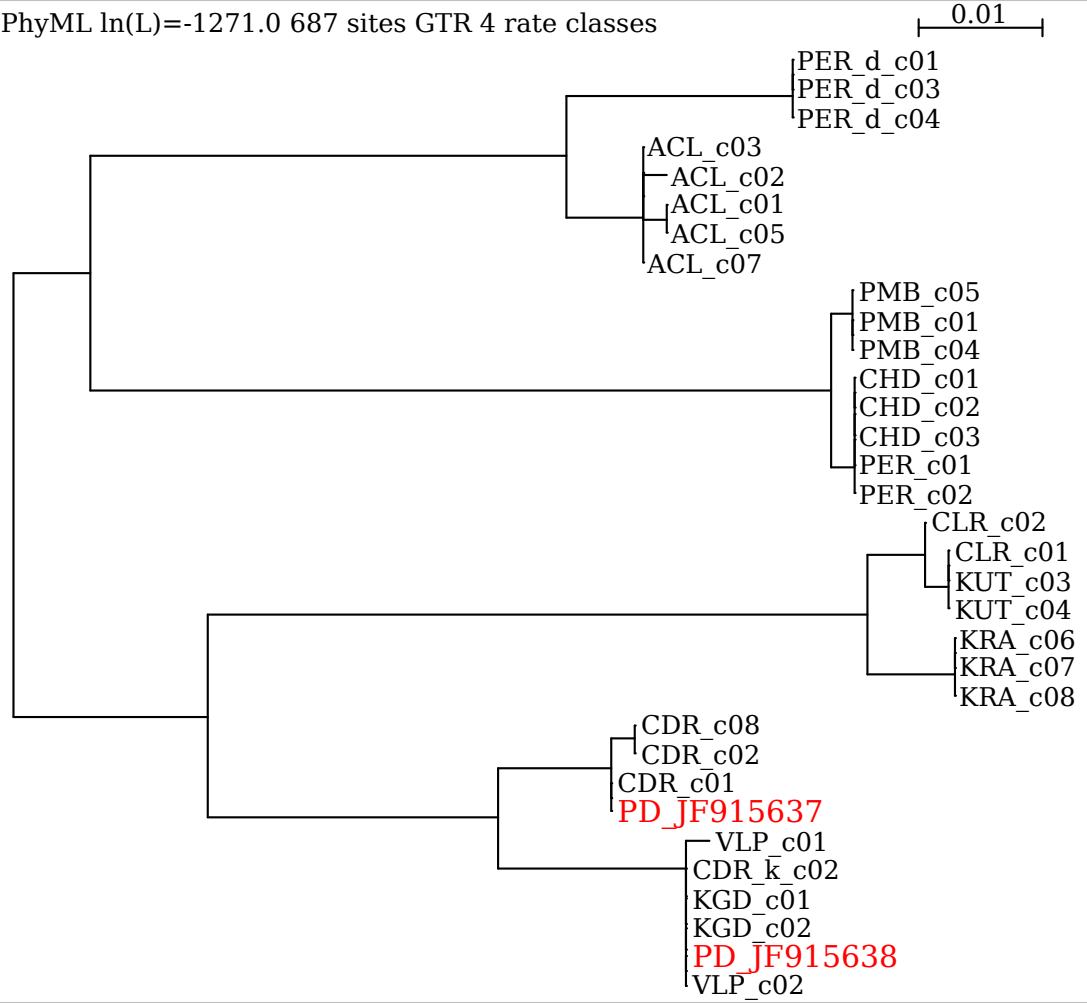

Supplement: Figure S8 — Tree showing the phylogenetic position of sequences from traded specimen. Tree showing the phylogenetic position of sequences generated for an earlier study [12] on aquarium trade. The sequences belong to two different evolutionarily distinct lineages CHD (PD_JF915637) and VLP-KGD (PD_JF915638), which are known to be the most heavily collected locations for RLTB trade. (PDF) [file pone.0069741.s008.pdf]
