# Supplementary material for: Morphological and Genetic Evidence for Multiple Evolutionary Distinct Lineages in the Endangered and Commercially Exploited Red Lined Torpedo Barbs Endemic to the Western Ghats of India
Source: PLoS One. 2013 Jul 22;8(7):e69741. doi: 10.1371/journal.pone.0069741 (PMC3718778; doi:10.1371/journal.pone.0069741)
Supplement: Table S1 — Analysis of Variance of size adjusted characters. (PDF) [file pone.0069741.s009.pdf]

Table S1. Analysis of Variance of size adjusted characters.

Characters	$F_{11,83}$	*P*
As %SL		
Head Length	18.544	< 0.0001
Body depth at dorsal	16.223	< 0.0001
Body depth at anus	17.750	< 0.0001
Body width at anus	5.632	< 0.0001
Pre Dorsal length	9.243	< 0.0001
Dorsal origin to caudal origin	6.428	< 0.0001
Pre Pectoral Length	4.493	< 0.0001
Pre Pelvic Length	7.907	< 0.0001
Pre anus length	11.417	< 0.0001
Pre anal length	14.178	< 0.0001
Length of caudal peduncle	4.399	< 0.0001
Depth of caudal peduncle	5.999	< 0.0001
Dorsal fin length	5.323	< 0.0001
Pectoral fin length	12.549	< 0.0001
Pelvic fin length	5.789	< 0.0001
Anal fin length	7.655	< 0.0001
Anal fin base	6.048	< 0.0001
As %HL		
Head Depth	10.805	< 0.0001
Head Width	3.078	0.002
Eye Diameter	6.270	< 0.0001
Snout Length	7.279	< 0.0001
Eye to nostril	4.384	< 0.0001
Posterior border of the eye to operculum	2.723	0.005
Inter orbital space	6.244	< 0.0001
