# Supplementary material for: Morphological and Genetic Evidence for Multiple Evolutionary Distinct Lineages in the Endangered and Commercially Exploited Red Lined Torpedo Barbs Endemic to the Western Ghats of India
Source: PLoS One. 2013 Jul 22;8(7):e69741. doi: 10.1371/journal.pone.0069741 (PMC3718778; doi:10.1371/journal.pone.0069741)
Supplement: Table S2 — MANOVA/CVA loadings for the first three canonical axes. (PDF) [file pone.0069741.s010.pdf]

Table S2. MANOVA/CVA loadings for the first three canonical axes

Variable	Axis 1	Axis 2	Axis 3
Head Length	-1.0364	-0.3378	0.5413
Body depth at dorsal	0.4461	-0.4208	-0.3340
Body depth at anus	0.4143	0.3288	-0.6828
Body width at dorsal	-0.0005	-0.2239	-0.0034
Body width at anus	-0.4787	0.0597	0.3816
Dorsal origin to caudal origin	0.1388	0.0651	-0.0706
Pre Pectoral Length	-0.0115	-0.5478	-0.0668
Pre Pelvic Length	0.2428	0.3370	-0.1624
Pre anus length	-0.1227	-0.0760	-0.4543
Pre anal length	-0.5179	0.4469	0.1920
Length of caudal peduncle	0.0809	-0.2017	0.6069
Depth of caudal peduncle	0.7643	0.1929	-0.2190
Dorsal fin length	-0.0285	-0.0981	-0.4304
Pectoral fin length	0.0639	-0.8844	0.1344
Pelvic fin length	-0.2806	0.3896	0.2120
Anal fin length	0.7158	-0.4653	-0.1788
Anal fin base	-0.1213	0.2696	0.9329
Head Depth	0.0619	0.0274	0.1190
Head Width	-0.0654	-0.1287	-0.0967
Eye Diameter	-0.1283	-0.0016	-0.0499
Snout Length	-0.0750	0.0785	0.2402
Eye to nostril	-0.1422	-0.1402	-0.1485
Posterior border of the eye to operculum	-0.0285	0.0383	0.0150
Inter orbital space	-0.1294	-0.0948	0.1986
