# Supplementary material for: Morphological and Genetic Evidence for Multiple Evolutionary Distinct Lineages in the Endangered and Commercially Exploited Red Lined Torpedo Barbs Endemic to the Western Ghats of India
Source: PLoS One. 2013 Jul 22;8(7):e69741. doi: 10.1371/journal.pone.0069741 (PMC3718778; doi:10.1371/journal.pone.0069741)
Supplement: Table S3 — Discriminant functions for the 12 populations (PDF) [file pone.0069741.s011.pdf]

Table S3. Discriminant functions for the 12 populations

	Populations north of Palakkad gap							Populations south of Palakkad gap			
	CDR	CDRK	VLP	KGD	CLR	KUT	KRA	CHD	PER	PERD	PMB
Intercept	-6201.417	-6061.030	-6152.064	-6255.463	-6201.647	-6028.999	-6070.577	-5937.168	-6038.358	-5801.318	-6124.918
%SL											
Head Length	116.966	109.763	110.032	117.050	107.860	105.221	115.580	109.290	111.949	109.610	115.796
Body depth at dorsal	8.321	10.444	10.020	9.073	7.634	7.420	9.025	11.581	17.658	17.834	13.766
Body depth at anus	-18.599	-15.827	-11.040	-18.477	-13.905	-9.849	-15.127	-13.965	-14.779	-15.180	-19.939
Body width at dorsal	20.149	21.288	19.063	20.741	21.592	21.564	17.760	21.171	20.259	15.001	22.809
Body width at anus	9.591	6.393	7.776	9.896	6.685	5.073	8.743	4.399	2.658	3.568	6.887
Pre Dorsal length	12.236	11.450	13.039	12.493	14.091	12.927	12.404	10.279	9.611	10.057	8.655
Dorsal origin to caudal origin	26.672	25.995	26.669	26.561	26.624	26.628	26.178	28.112	28.348	28.288	26.565
Pre Pectoral Length	3.635	6.342	2.123	5.097	4.050	6.288	0.865	3.696	6.592	4.131	8.097
Pre Pelvic Length	-23.755	-23.002	-20.606	-24.742	-21.089	-21.232	-21.262	-21.101	-22.194	-19.730	-24.645
Pre anus length	34.842	34.622	35.154	36.618	36.098	36.600	31.326	32.546	33.906	29.768	33.541
Pre anal length	55.328	54.361	53.342	54.056	54.694	52.239	55.193	51.053	48.920	49.969	51.713
Pelvic to anus distance	-7.732	-6.470	-5.866	-7.977	-7.192	-6.518	-5.890	-5.252	-4.064	-0.310	-8.381
Length of caudal peduncle	18.109	18.300	15.944	17.492	16.862	16.661	19.075	18.905	17.870	21.191	22.018
Depth of caudal peduncle	9.541	5.572	11.587	8.075	10.065	6.552	10.066	15.336	12.804	6.802	9.616
Dorsal fin length	-7.998	-7.534	-7.303	-8.187	-6.995	-6.521	-9.911	-9.669	-7.328	-10.264	-9.016
Dorsal fin base	-24.671	-23.253	-22.832	-24.419	-24.185	-27.950	-23.020	-27.687	-28.325	-28.940	-28.126
Pectoral fin length	23.900	30.457	25.799	28.567	24.806	27.047	24.066	27.151	25.691	22.034	31.348
Pelvic fin length	-16.181	-16.838	-18.018	-16.522	-17.098	-15.964	-13.481	-16.619	-17.703	-12.778	-17.678
Anal fin length	-18.097	-16.276	-15.531	-19.047	-17.098	-14.204	-17.388	-12.102	-11.352	-16.695	-11.049
Anal fin base	3.583	-1.018	-0.017	2.786	0.232	-5.268	5.231	5.614	0.979	7.445	6.406
%HL											
Head Depth	12.149	12.121	11.756	11.723	11.678	11.607	12.400	13.029	12.628	13.485	12.583
Head Width	10.765	9.891	10.440	10.588	9.863	9.607	10.956	9.834	11.163	9.265	11.763
Eye Diameter	9.848	8.773	9.433	10.274	8.993	9.478	10.380	9.232	10.506	11.470	10.138
Snout Length	5.131	3.997	3.212	4.679	4.060	3.092	3.795	4.872	3.917	5.447	4.467
Eye to nostril	14.415	15.145	14.562	15.665	15.600	15.520	12.807	13.968	15.077	11.969	14.346
Posterior border of the eye to operculum	-4.104	-4.440	-4.169	-4.202	-4.019	-3.655	-4.102	-4.113	-4.539	-5.019	-4.621
Barbel length	-1.338	-0.317	-0.181	-1.296	0.398	0.674	-2.068	-2.303	-2.139	-2.374	-2.893
Inter orbital space	12.229	11.595	10.484	12.206	10.636	10.601	12.090	11.527	11.157	12.091	13.513
