# Supplementary material for: Morphological and Genetic Evidence for Multiple Evolutionary Distinct Lineages in the Endangered and Commercially Exploited Red Lined Torpedo Barbs Endemic to the Western Ghats of India
Source: PLoS One. 2013 Jul 22;8(7):e69741. doi: 10.1371/journal.pone.0069741 (PMC3718778; doi:10.1371/journal.pone.0069741)
Supplement: Table S4 — Detailed results of the GMYC methods implemented for the cytb ultrametric tree and the concatenated ultrametric tree, the species distinction made is displayed in the Figure 3b in the main text. (PDF) [file pone.0069741.s012.pdf]

Table S4: Detailed results of the GMYC methods implemented for the *cytb* ultrametric tree and the Concatenated ultrametric tree, the species distinction made is displayed in the Figure 3b in the main text.

Result of GMYC species delimitation using the Concatenated ultrametric tree:

Method: single

Likelihood of null model:	171.6296
Maximum likelihood of GMYC model:	187.7243
Likelihood ratio:	32.18945
Result of LR test:	4.773749e-07***
Number of ML clusters:	**7**
Confidence interval:	7-8
Number of ML entities:	27
Confidence interval:	11-27
Threshold time:	-0.0001780219

Method: multiple

Likelihood of null model:	171.6296
Maximum likelihood of GMYC model:	189.5913
Likelihood ratio:	35.92345
Result of LR test:	9.838917e-07***
Number of ML clusters:	9 (clusters reported in figure 2d)
Confidence interval:	8-9
Number of ML entities:	15
Confidence interval:	12-19
Threshold time:	-0.03990777
	-0.01748675
	-0.004642341

Comparison of single and multiple threshold GMYC

Chi-square = 3.7340, df = 6, P = 0.7126

Result of GMYC species delimitation using the CYTb ultrametric tree:

Method: single

Likelihood of null model:	187.1918
Maximum likelihood of GMYC model:	209.7168
Likelihood ratio:	45.05
Result of LR test:	9.029038e-10***
Number of ML clusters:	8
Confidence interval:	8-8
Number of ML entities:	23
Confidence interval:	23-23
Threshold time:	-0.0002175

Method: multiple

Likelihood of null model:	187.1918
Maximum likelihood of GMYC model:	198.8942
Likelihood ratio:	23.40481
Result of LR test:	0.000282415***
Number of ML clusters:	6 (clusters reported in figure 2d)
Confidence interval:	6-6
Number of ML entities:	10
Confidence interval:	9-10
Threshold time:	-0.1688247
	-0.008929472
	-0.005099265

Comparison of single and multiple threshold GMYC

Chi-square = 21.6452, df = 6, P = 0.0014
