# Supplementary material for: Morphological and Genetic Evidence for Multiple Evolutionary Distinct Lineages in the Endangered and Commercially Exploited Red Lined Torpedo Barbs Endemic to the Western Ghats of India
Source: PLoS One. 2013 Jul 22;8(7):e69741. doi: 10.1371/journal.pone.0069741 (PMC3718778; doi:10.1371/journal.pone.0069741)
Supplement: Table S5 — Table showing the divergence times for the RLTB's internal node number are in the first column which follows the Figure S5. (PDF) [file pone.0069741.s013.pdf]

Table S5: Table showing the divergence times for the RLTB's internal node number are in the first column which follows the Figure S5.

Node	95% confidence interval (x100 Ma)		mean (Ma)
n44	1.4385	–2.3455	177.78
n45	1.3785	–2.292	172.79
n46	1.0906	–1.9135	141.75
n47	0.8574	–1.5553	113.95
n48	0.6956	–1.2808	93.38
n49	0.4074	–0.8001	56.99
n50	0.2817	–0.5913	41.01
n51	0.0206	–0.0769	4.23
n52	0.0048	–0.0317	1.47
n53	0.003	–0.0228	1.02
n54	0.0018	–0.0165	0.71
n55	0.0009	–0.0121	0.47
n56	0.0003	–0.0089	0.29
n57	0.0035	–0.0271	1.2
n58	0.002	–0.0193	0.81
n59	0.001	–0.0139	0.54
n60	0.0004	–0.0099	0.33
n61	0.0632	–0.1767	10.82
n62	0.01	–0.0488	2.45
n63	0.0031	–0.0255	1.13
n64	0.0015	–0.018	0.74
n65	0.0001	–0.0076	0.21
n66	0.002	–0.0235	0.98
n67	0.0007	–0.0155	0.56
n68	0.0036	–0.0352	1.47
n69	0.0001	–0.0129	0.35
n70	0.2859	–0.6125	42.14
n71	0.0348	–0.1177	6.73
n72	0.0056	–0.0406	1.86
n73	0.0001	–0.0121	0.33
n74	0.003	–0.028	1.18
n75	0.0014	–0.0179	0.72
n76	0.0005	–0.0121	0.43
n77	0.1067	–0.2812	17.81
n78	0.009	–0.0485	2.38
n79	0.0032	–0.0272	1.2
n80	0.0006	–0.0145	0.5
n81	0.0001	–0.0172	0.47
n82	0.0055	–0.0421	1.9
n83	0.0009	–0.0199	0.7
n84	0.4691	–0.9559	67.87
n85	0.7776	–1.4534	105.25
