# Supplementary material for: Morphological and Genetic Evidence for Multiple Evolutionary Distinct Lineages in the Endangered and Commercially Exploited Red Lined Torpedo Barbs Endemic to the Western Ghats of India
Source: PLoS One. 2013 Jul 22;8(7):e69741. doi: 10.1371/journal.pone.0069741 (PMC3718778; doi:10.1371/journal.pone.0069741)
Supplement: Table S6 — Confusion matrix for group identity based on discriminant functions. Populations in the row are original identities. Populations in the column are predicted identities. Diagonal elements indicate correct prediction of group identity. Off diagonal elements show wrong predictions. (PDF) [file pone.0069741.s014.pdf]

Table S6. Confusion matrix for group identity based on discriminant functions. Populations in the row are original identities. Populations in the column are predicted identities. Diagonal elements indicate correct prediction of group identity. Off diagonal elements show wrong predictions

from \ to	CDR	CDRK	VLP	KGD	CLR	KUT	KRA	CHD	PER	PERD	PMB	ACL	Total	% correct
CDR	**10**	0	0	0	0	0	0	0	0	0	0	0	10	100.00
CDRK	0	**9**	0	0	***1***	0	0	0	0	0	0	0	10	90.00
VLP	0	0	**10**	0	0	0	0	0	0	0	0	0	10	100.00
KGD	0	0	0	**10**	0	0	0	0	0	0	0	0	10	100.00
CLR	0	0	0	0	**11**	0	0	0	0	0	0	0	11	100.00
KUT	0	0	0	0	0	**2**	0	0	0	0	0	0	2	100.00
KRA	0	0	0	0	0	0	**5**	0	0	0	0	0	5	100.00
CHD	0	0	0	0	0	0	0	**10**	0	0	0	0	10	100.00
PER	0	0	0	0	0	0	0	0	**10**	0	0	0	10	100.00
PERD	0	0	0	0	0	0	0	0	0	**3**	0	0	3	100.00
PMB	0	0	0	0	0	0	0	0	0	0	**9**	0	9	100.00
ACL	0	0	0	0	0	0	0	0	0	0	0	**5**	5	100.00
Total	10	9	10	10	12	2	5	10	10	3	9	5	95	98.95
