# Supplementary material for: Morphological and Genetic Evidence for Multiple Evolutionary Distinct Lineages in the Endangered and Commercially Exploited Red Lined Torpedo Barbs Endemic to the Western Ghats of India
Source: PLoS One. 2013 Jul 22;8(7):e69741. doi: 10.1371/journal.pone.0069741 (PMC3718778; doi:10.1371/journal.pone.0069741)
Supplement: Table S7 — Samples used and sampling sites: a) Samples procured from aquarium collectors, corresponding river systems and number of samples used; b) List of sampling sites from where we collected samples directly, corresponding river systems and number of samples used. (PDF) [file pone.0069741.s015.pdf]

Table S7

Samples procured from aquarium collectors, corresponding river systems and number of samples used

River System@	Collection Sites	n- MOR	n-MOL
Chandragiri/CDR[1]	Sullya	10	3
Chandragiri/CDRK[1]	Kottody, Nagapattinam	10	1
Karyangode/KGD[1]	Cherupuzha	10	2
Valapattanam/VLP[2]	Iritty	10	2
Bharatapuzha/KRA[3]	Kanjirapuzha	5	2

[1] Collected in 2005 and 2006
[2] Collected in 2010
[3] Collected in 2008
n-MOR: number of samples used for morphological analysis.
n-MOL: number of samples used for molecular analysis.

List of sampling sites from where we collected samples directly, corresponding river systems and number of samples used

River System@	Collection Sites	n- MOR	n-MOL
Kuttyadi/KUT[$, 1]	Chathangothunada	2	2(1)
Chaliyar/CLR[$, 2]	Chalipuzha, Pullooranpara	11(7)	2
Chalakudy/CHD[#, 3]	Athirapilly, Vettilapara	10(2)	3(1)
Periyar/PER[#, 3]	Paniyeli, Pooyamkutty	10(6)	2
Periyar/PERD[#, 3]	Pooyamkutty	3	3(2)
Pampa/PMB[#, 3]	Angel Valley, Azhutha, Koruthodu	9(2)	6(4)
Achankovil/ACL[#, 3]	Mukkada, Chuttipara, Kadakkola	5(3)	8(3)

[1]Collected in 2008
[2] Collected in 2007 and 2009
[3] Collected in 2010
[$]no permits were required
[#]permits were required

n-MOR: number of samples used for morphological analysis; numbers in parenthesis denotes number of fishes released back after measurements/sampling.
n-MOL: number of samples used for molecular analysis; numbers in parenthesis denotes number of fishes released back after measurements/sampling.
