# Supplementary material for: Morphological and Genetic Evidence for Multiple Evolutionary Distinct Lineages in the Endangered and Commercially Exploited Red Lined Torpedo Barbs Endemic to the Western Ghats of India
Source: PLoS One. 2013 Jul 22;8(7):e69741. doi: 10.1371/journal.pone.0069741 (PMC3718778; doi:10.1371/journal.pone.0069741)
Supplement: Table S8 — Genbank details of the sequences used in the study and codes for the trace files of sequences uploaded at figshare (http://dx.doi.org/10.6084/m9.figshare.95635). (PDF) [file pone.0069741.s016.pdf]

Table S8. Genbank details of the sequences used in the study and codes for the trace files of sequences uploaded at figshare (http://dx.doi.org/10.6084/m9.figshare.95635).

Sl.No	Trace file ID*	Sequence ID in Paper/voucher codes	Accession No. COI	Accession No. CytB	Source
1		CDR01	GQ247550	GQ247558	NCBI
2	023	CDR02	GQ247551 (NCBI)	JX470422	This study+NCBI
3	109	CDR03	JX462903	JX462890	This study
4	CDRK2	CDRK	JX462866	JX462876	This study
5		KGD01	GQ247554	GQ247559	NCBI
6	035	KGD02	JX470428	JX470423	This study
7		VLP01	GQ247555	GQ247561	NCBI
8	005	VLP02	JX470427	JX470421	This study
9		CLR01	GQ247552	GQ247560	NCBI
10	171	CLR02	GQ247553 (NCBI)	JX470426	This study+NCBI
11		CHD01	GQ247549	GQ247556	NCBI
12	043	CHD02	JX481180	JX470424	This study
13	49	CHD03	JX462904	JX462891	This study
14	052	PER01	JX481181	JX470425	This study
15	053	PER02	JX470429	GQ247557(NCBi)	This study+NCBI
16	215	PERD03	JX462905	JX462892	This study
17	288	PERD04	JX462906	JX462893	This study
18	289	PERD05	JX462907	JX462894	This study
19	301	PMB01	JX462908	JX462895	This study
20	305	PMB02	JX462909	JX462896	This study
21	306	PMB03	JX462910	JX462897	This study
22	ACL1	ACL01	JX462898	JX462898	This study
23	ACL2	ACL02	JX462899	JX462886	This study
24	ACL3	ACL03	JX462900	JX462887	This study
25	ACL5	ACL05	JX462901	JX462888	This study
26	ACL7	ACL07	JX462902	JX462889	This study
27	KRA6	ACL06	JX462867	JX462877	This study
28	KRA7	KRA07	JX462868	JX462878	This study
29	KRA8	KRA08	JX462869	JX462879	This study
30	KUT3	KUT03	JX462874	JX462883	This study
31	KUT4	KUT04	JX462875	JX462884	This study
32	MLA1	PMB11	JX481187	JX481182	This study
33	MLA2	PMB12	JX481185	JX481183	This study
34	MLA3	PMB13	JX481186	JX481184	This study
35	ACH2	ACL8	JX481188	JX470430	This study
36	ACH3	ACL9	JX481189	JX470431	This study

* the code (recognizable in the filename) for the sequence trace files uploaded at figshare http://dx.doi.org/10.6084/m9.figshare.95635
